# *Anaplasma*, *Bartonella*, and *Rickettsia* infections in Daurian ground squirrels (*Spermophilus dauricus*), Hebei, China

**DOI:** 10.3389/fmicb.2024.1359797

**Published:** 2024-03-28

**Authors:** Jing Xue, Si-Si Chen, Ze-Yun Xu, Fang-Ni Wang, Jiangli Wang, Danhong Diao, Luanying Du, Guang-Cheng Xie, Wen-Ping Guo

**Affiliations:** ^1^College of Basic Medicine, Chengde Medical University, Chengde, China; ^2^Chengde Center for Disease Control and Prevention, Chengde, China

**Keywords:** Daurian ground squirrel, *Anaplasma*, *Bartonella*, *Rickettsia*, genetic diversity

## Abstract

Rodents have been confirmed as hosts of various vector-borne zoonotic pathogens and are important for the maintenance of these microbes in nature. However, surveillance for zoonotic pathogens is limited for many wild rodent species in China, so our knowledge of pathogen ecology, genetic diversity, and the risk of cross-species transmission to humans is limited. In this study, 165 spleen samples of Daurian ground squirrels (*Spermophilus dauricus*) were collected from Weichang Manchu and the Mongolian Autonomous County of Hebei Province, China, and *Rickettsia*, *Bartonella*, and *Anaplasma* were identified by DNA detection using polymerase chain reaction (PCR). Sequence analysis identified eight bacterial pathogens: *R. raoultii*, *R. sibirica*, *Candidatus* R. longicornii, *B. washoensis*, *B. grahamii*, *B. jaculi*, *A. capra*, and *Candidatus* Anaplasma cinensis. Co-infection of *B. grahamii* and *R. raoultii* in one sample was observed. Our results demonstrated the genetic diversity of bacteria in Daurian ground squirrels and contributed to the distribution of these pathogens. Six species, *A. capra*, *R. raoultii*, *R. sibirica*, *Candidatus* R. longicornii, *B. washoensis*, and *B. grahamii*, are known to be pathogenic to humans, indicating a potential public health risk to the local human population, especially to herders who frequently have close contact with Daurian ground squirrels and are thus exposed to their ectoparasites.

## Introduction

Rodents are the most diverse taxa of mammals (Huchon et al., 2002; Wilson and Reeder, 2005) and serve as reservoirs of many important zoonotic pathogens (Meerburg et al., 2009; Han et al., 2015). These pathogens consist of bacteria, viruses, and parasites. They comprise 66 identified zoonotic pathogens harbored by a minimum of 217 rodent species as reservoirs. Additionally, at least 79 rodent species can host between 2 and 11 zoonotic causative agents (Han et al., 2015). In recent years, an increasing number of viruses have been discovered by next-generation sequencing technology in rodents, some of which possess zoonotic potential (Chen et al., 2023). Furthermore, most of these zoonotic pathogens are indirectly transmitted from rodents to humans via arthropod vectors (Meerburg et al., 2009).

*Rickettsia* and *Anaplasma* (Order Rickettsiales) are obligate intracellular bacteria that are transmitted by ticks (Darby et al., 2007). Some *Rickettsia* species have been detected in rodents, although the role that rodents play in their life cycle is unclear (Schex et al., 2011; Kuo et al., 2015; Lu et al., 2019). Unlike *Rickettsia*, *Anaplasma* cannot be transmitted transovarially in ticks; therefore, vertebrate mammals such as rodents are required to complete their life cycle (Battilani et al., 2017). Bacteria belonging to the genus *Bartonella* include several zoonotic species that can be transmitted from animals to humans through the bites of infected bloodsucking arthropods (Deng et al., 2012), as well as through the scratch of an infected cat or by contact with the infected feces of a vector (Krügel et al., 2022). Although *Bartonella* DNA has been detected in ticks, the role of ticks as vectors in the transmission of *Bartonella* spp. remains unclear (Telford and Wormser, 2010). As the natural hosts, more than half of all *Bartonella* species, including human pathogens, have been identified in rodents to date (Jian et al., 2022).

The Daurian ground squirrel (*Spermophilus dauricus*) may be a potential reservoir of tick-borne pathogens. In China, a tentative *Ehrlichia* species was identified from this ground squirrel in Inner Mongolia (Li et al., 2023a). In addition, *B. rochalimae* and *B. washoensis* were detected in Inner Mongolia (Li et al., 2023b). Except for the three above-mentioned bacterial species and *Y. pestis*, no other vector-borne intracellular bacteria have been found in Daurian ground squirrels. In our preliminary studies, herders parasitized by ticks were found in the Bashang area of Weichang Manchu and the Mongolian Autonomous County of Hebei Province, China. Therefore, when herders enter the habitat of Daurian ground squirrels, they may come into contact with the rodents and be exposed to their ectoparasites. A previous study showed that intracellular bacterial pathogens, including *R. raoultii* and *Candidatus* R. tarasevichiae, were identified in 26 ticks collected from humans (Xue et al., 2023) in Weichang County, as well as *A. ovis* with zoonotic potential. Therefore, to better understand the genetic diversity of intracellular bacterial pathogens, Daurian ground squirrels were collected from the Bashang area to screen for the presence of neglected vector-borne *Anaplasma*, *Bartonella*, and *Rickettsia*. The results will help to assess the potential transmission risk of these pathogens to humans.

## Materials and methods

### Sample collection of rodents and DNA extraction

**Figure 1:**
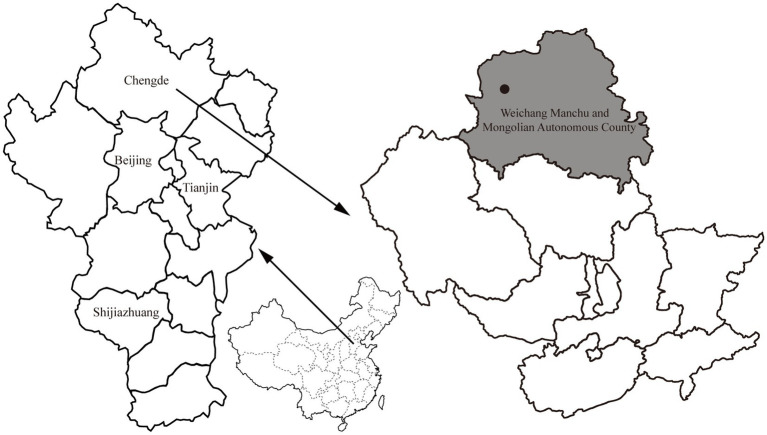
Map with the location of the sampling site (black circle) in Weichang Manchu and Mongolian Autonomous County (shown in gray), Hebei, China.

From April to October 2021, rodents were captured alive using baited cages with a treadle release mechanism from one sampling site (approximately 3 km^2^) located in the Bashang area of Weichang Manchu and Mongolian Autonomous County, Hebei Province, China (Figure 1). The cages were set at night and then retrieved the following day. Two hundred cages were used in each trapping session, and two or three trapping sessions were conducted each month. Based on the standard taxonomic characteristics (Wang, 2003), the rodents were first identified morphologically at the species level. After species identification, the sampled rodents were euthanized with an isoflurane overdose before surgery to minimize suffering. Spleen tissue samples were aseptically collected from each individual and stored on dry ice. A volume of 200 μL of spleen tissue suspension was prepared by homogenizing a 30 mg sample in 200 μL of TL buffer. DNA was isolated from the spleen suspension of each rodent using a Tissue DNA Kit (Omega, Norcross, GA, United States) according to the manufacturer’s protocol. The extracted DNA was eluted in 80 μL of elution buffer and stored at −20°C before pathogen detection and confirmation of rodent species by molecular methods. All samples tested negative for *Yersinia pestis* by detecting immunoglobulin (IgG) antibodies against the F1 antigen and *caf1* and *pla* genes before screening for other pathogens (Zhao et al., 2017). The rodent species was confirmed by sequence analysis of the mitochondrial cytochrome b (mt-*cyt b*) gene (Guo et al., 2013). This study was approved by the Scientific Ethics Committee of the Chengde Medical University (No. 202004).

### Molecular detection of *Rickettsia*, *Anaplasma*, and *Bartonella*

*Rickettsia*, *Anaplasma*, and *Bartonella* were detected for the presence of DNA using polymerase chain reaction (PCR). *Rickettsia* was screened using semi-nested PCR targeting a conserved region of the outer membrane protein (*ompA*) (Ishikura et al., 2003). The DNA of *Bartonella* was detected by amplifying a fragment of the citrate synthase (*gltA*) gene (Jian et al., 2022). *Anaplasma* was detected by semi-nested PCR using the primer pairs fD1/Eh-out2 and fD1/Eh-gs1 for primary and secondary rounds, targeting the 16S rRNA (Weisburg et al., 1991; Wen et al., 2002). In addition, the DNA of *Candidatus* Anaplasma cinensis was detected to confirm its presence by amplifying the partial *gltA* gene using primers designed for this study. The partial *groEL* gene of *Candidatus* Anaplasma cinensis was amplified to better characterize its genetic characteristics. The primers used in the present study are listed in Table 1. For *Candidatus* A. cinensis, the PCR for the primary round of semi-nested PCR was performed in a 20 μL reaction volume containing 10 μL of Premix Taq (Takara, Dalian, China), 1.6 μL of DNA, 1.0 μL of each primer (10 pmol), and 5.4 μL of water. The secondary round consisted of a final volume of 50 μL that contained 25 μL of PCR mixture Premix Taq (Takara, Dalian, China), 3 μL of the first round PCR products, 2 μL of each primer (10 pmol), and 18 μL of water. The same thermal cycling conditions were used for both rounds and were as follows: pre-denaturation at 94°C for 5 min; 35 cycles of denaturation at 94°C for 40 s; annealing at 56°C for 40 s; elongation at 72°C for 1 min; and a final extension at 72°C for 7 min. Double-distilled water was used as a negative control. In addition, positive controls were included in the reactions using DNA deposited in our laboratory.

**Table 1:** Primer sequences used in this study.

Pathogens	Target gene	Primer	Oligonucleotide sequences (5′–3′)	Size (bp)	References
*Rickettsia*	*ompA*	Rr190k.70p	TGGCGAATATTTCTCCAAAA (+)	532	Ishikura et al. (2003)
		Rr190k.720n	TGCATTTGTATTACCTATTGT (−)		
		Rr190k.602n	AGTGCAGCATTCGCTCCCCCT (−)		
*Bartonella*	*gltA*	Bar-gltA-FM	GCHGATCAYGARCAAAATGC (+)	476	Jian et al. (2022)
		Bar-gltA-R1	CYTCRATCATTTCTTTCCAYTG (−)		
		Bar-gltA-R2	GCAAAVAGAACMGTRAACAT (−)		
*Candidatus* Anaplasma cinensis	*gltA*	Platys-glta-F	GATGTGTGGGAAAGAAGAAAT (+)	641	This study
		Pglt-R1	TCATGRTCTGCATGCATKATG (−)		Guo et al. (2019)
		Pglt-R2	CATGCATKATGAARATCGCRT (−)		
	*groEL*	Platys-groEL-F1	GAGTATTAASCCTGAGGAAC (+)	439	This study
		Platys-groEL-F2	AGAGTGCATCGCAATGTAAT (+)		
		Platys-groEL-R	AGGAATATGTAYGGRTTYTC (−)		
*Anaplasma*	16S rRNA	fD1	AGAGTTTGATCCTGGCTCAG (+)	639	Weisburg et al. (1991) and Wen et al. (2002)
		Eh-out2	AGTAYCGRACCAGATAGCCGC (−)		
		Eh-gs2	CTAGGAATTCCGCTATCCTCT (−)a		

The PCR products were separated by electrophoresis using 1% agarose gels, and the bands were observed after staining. The PCR products of the expected size were purified using a MiniBEST Agarose Gel DNA Extraction Kit Ver.4.0 (TaKaRa, Dalian, China). Due to the sequences being less than 700 bp, the amplicons were bidirectionally sequenced using the primers for the secondary round of the PCR after purification. If double peaks were visible on the sequencing chromatograms, the amplicons were cloned into pMD19-T vectors (Takara, Dalian, China) for further sequencing with the universal primers RV-M and M13-47 (Sangon, Beijing, China).

### Nucleotide sequence identity and phylogenetic analyses

All the newly generated sequences were searched against the GenBank database using the Basic Local Alignment Search Tool (BLAST). The nucleotide sequence identities between the sequences in this study and reference sequences were determined using the MegAlign program in Lasergene (Burland, 2000). A maximum likelihood (ML) tree used to identify the pathogens by species was reconstructed using PhyML 3.0 (Guindon et al., 2010) based on the optimal nucleotide substitution model (GTR + Γ + I) estimated by MEGA 6.0.6 (Tamura et al., 2013). The reliability of the tree was evaluated by bootstrap analysis with 1,000 replicates.

### Statistical analysis

The differences between positive rates of *Anaplasma*, *Bartonella*, and *Rickettsia* infections in Daurian ground squirrels were compared using a *χ*^2^ test with a *p*-value of 0.05 as the threshold.

## Results

### Rodent sample collection

A total of 165 rodents were captured in Weichang Manchu and Mongolian Autonomous County, Hebei Province, China. Based on both morphological and molecular methods, all of the rodents were identified as Daurian ground squirrels, belonging to the family Sciuridae. The *cytb* gene sequences shared 99.3–100% nucleotide identity with each other and displayed 98.5–99.7% nucleotide identity with known corresponding sequences from Daurian ground squirrels in the GenBank database.

### Molecular detection of pathogens

After electrophoresis, 35 PCR products of expected size were subjected to sequencing, and the results were further subjected to BLAST searches. In total, 34 rodent samples tested positive for at least one causative agent, and one co-infection of two pathogens was found. Furthermore, eight pathogens were identified. Specifically, 13 rodent samples tested positive for the genus *Rickettsia*, with a positive rate of 7.9%, including 7 *R. raoultii*, 3 *R. sibirica*, and 3 *Candidatus* R. longicornii. Fifteen rodent samples tested positive for the genus *Bartonella*, with a positive rate of 9.1%, including 10 *B. washoensis*, 3 *B. grahamii*, and 2 *B. jaculi*. Co-infection of *B. grahamii* and *R. raoultii* was observed in one sample. Six samples tested positive for *Candidatus* A. cinensis, with a positive rate of 3.6%. When using the primer pairs fD1/Eh-out2 and fD1/Eh-gs1 targeting the 16S rRNA of the genus *Anaplasma*, none of the six samples mentioned above tested positive. Instead, an additional sample yielded a positive result. After sequencing, BLASTn analysis based on this partial 16S rRNA sequence indicated that this agent could be identified as *A. capra*, with a low positive rate of 0.6%. Unfortunately, we failed to get other genes. *Rickettsia* and *Bartonella* infections in Daurian ground squirrels had no significant difference (χ^2^ = 0.16, *p* = 0.69). The positive rate of *Bartonella* infection in Daurian ground squirrels was higher than that of *Anaplasma* (*χ*^2^ = 4.12, *p* = 0.04). However, no significant difference was observed between *Rickettsia* and *Anaplasma* infections (*χ*^2^ = 2.74, *p* = 0.10).

### Molecular characterization of identified vector-borne intracellular bacterial pathogens

**Figure 2:**
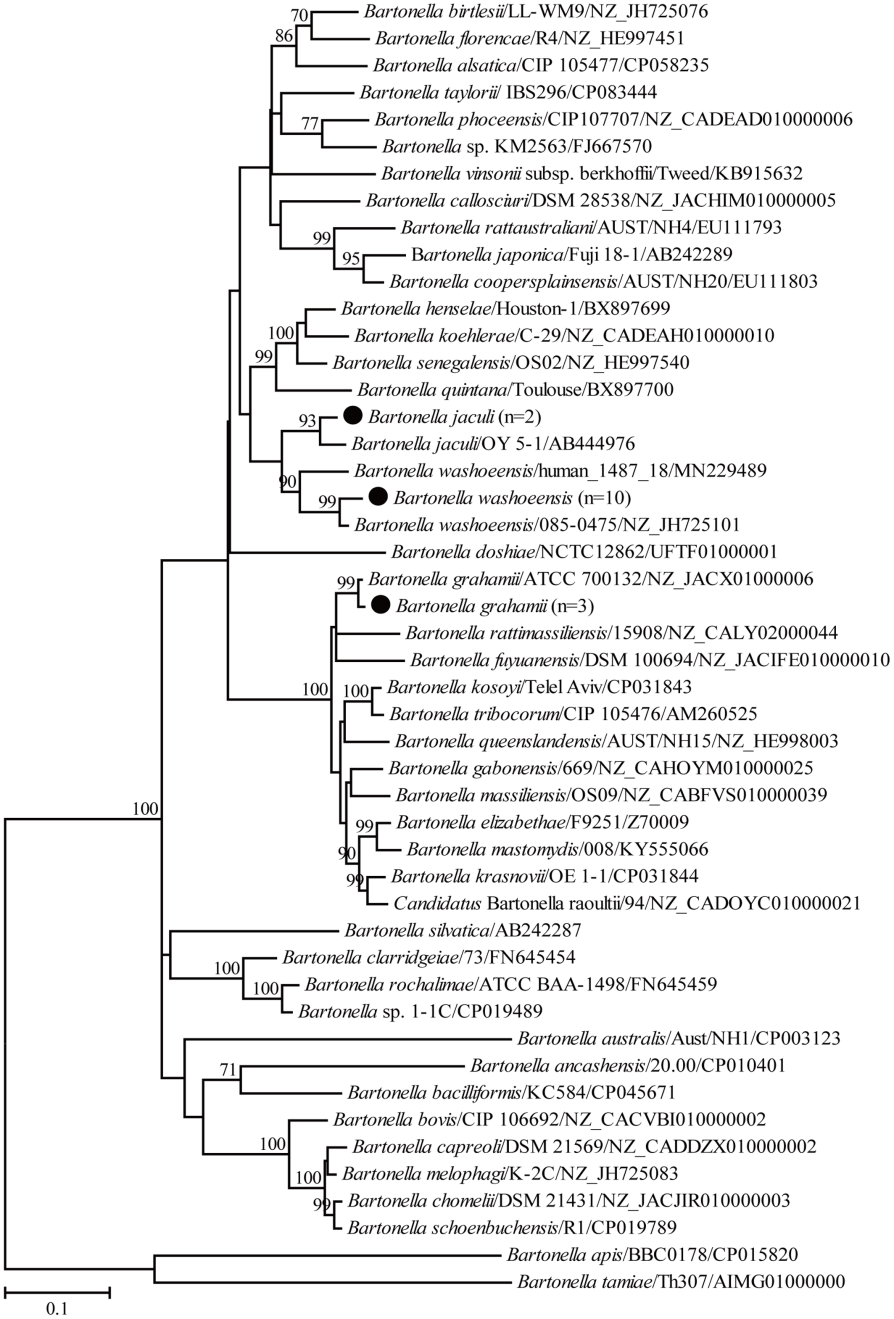
Phylogenetic tree based on the partial *gltA* gene sequences of the genus *Bartonella*. Numbers at each node indicate bootstrap values. The tree was mid-point rooted for clarity, and the scale bar represents the number of nucleotide substitutions per site. The representative sequence obtained in this study was used to reconstruct the tree and is marked with circles.

For the genus *Bartonella*, 10 partial *gltA* gene sequences of *B. washoensis* in this study shared 99.3–100% nucleotide identity with each other and the highest nucleotide identity of 99.7–100% with *Bartonella* sp. DR 1–1, also in Daurian ground squirrels from China (Inoue et al., 2009). Specifically, the *gltA* gene sequences recovered in this study shared 97.1–97.9% nucleotide identity with those of strain 085-0475 isolated from humans. In addition, the *gltA* gene sequences in this study shared 97.0–99.7% nucleotide identity with known ones of *B. washoensis* from ground squirrels and 96.4–98.8% nucleotide identity with known *B. washoensis* variants from other reservoirs. In the phylogenetic tree, all *B. washoensis* variants in this study clustered with the reference *B. washoensis* strain 085-0475, and then with *B. washoensis* isolate human_1487_18, also from humans (Figure 2). However, these newly generated sequences only shared 91.1–92.0% nucleotide identity with the *gltA* gene sequence of isolate human_1487_18. In addition, another three partial *gltA* gene sequences of *B. grahamii* in this study shared 99.1–99.8% nucleotide identity with each other, and 96.6–98.9% with known ones of *B. grahamii*. In the phylogenetic tree, all three newly generated *gltA* gene sequences clustered with those of *B. grahamii* (Figure 2). Two partial *gltA* gene sequences of *B. jaculi* recovered in this study shared 95.7% nucleotide identity with each other and 96.3–99.7% nucleotide identity with known ones of *B. jaculi*. In the phylogenetic tree, these two sequences clustered with that of a known *B. jaculi* isolate (Figure 2).

**Figure 3:**
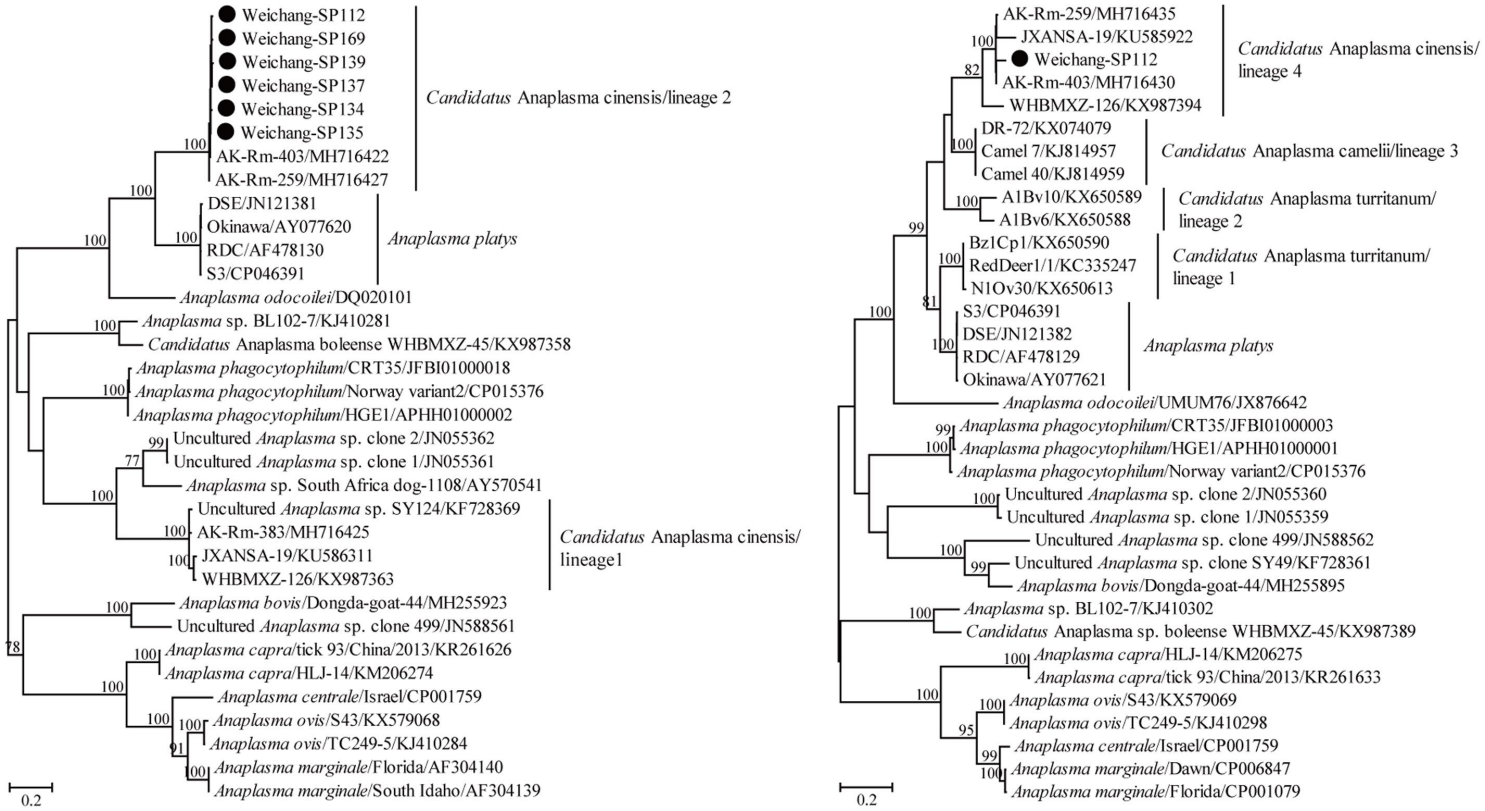
Phylogenetic tree based on the partial *gltA* (left) and *groEL* (right) gene sequences of the genus *Anaplasma*. Numbers at each node indicate bootstrap values. The tree was mid-point rooted for clarity, and the scale bar represents the number of nucleotide substitutions per site. The representative sequence obtained in this study was used to reconstruct the tree and is marked with circles.

The partial 16S rRNA gene sequence in this study presented a 100% similarity with some sequences of *A. capra* variants, suggesting a possible *A. capra* infection in Daurian ground squirrels. Using the primers designed in this study, *Candidatus* A. cinensis was detected by amplifying its *gltA* gene in six samples. All six of these partial *gltA* gene sequences in this study shared 99.0–99.7% nucleotide identity and shared 98.7–99.7% nucleotide identity with known ones of *Candidatus* A. cinensis. In addition, a partial *groEL* gene sequence was obtained and showed the highest nucleotide identity of 98.4% with that of known *Candidatus* A. cinensis variants. Moreover, the *groEL* and *gltA* gene sequences herein shared 82.8% and 74.0–74.7% nucleotide identity, respectively, with those of the *A. platys* reference strain S3. Consistently, these variants in the present study clustered with other known *Candidatus* A. cinensis rather than specific *A. platys*. Specifically, the *Candidatus* A. cinensis variants herein clustered within lineages 2 and 4 of Guo et al. (2019) for the *gltA* and *groEL* genes, respectively (Figure 3).

**Figure 4:**
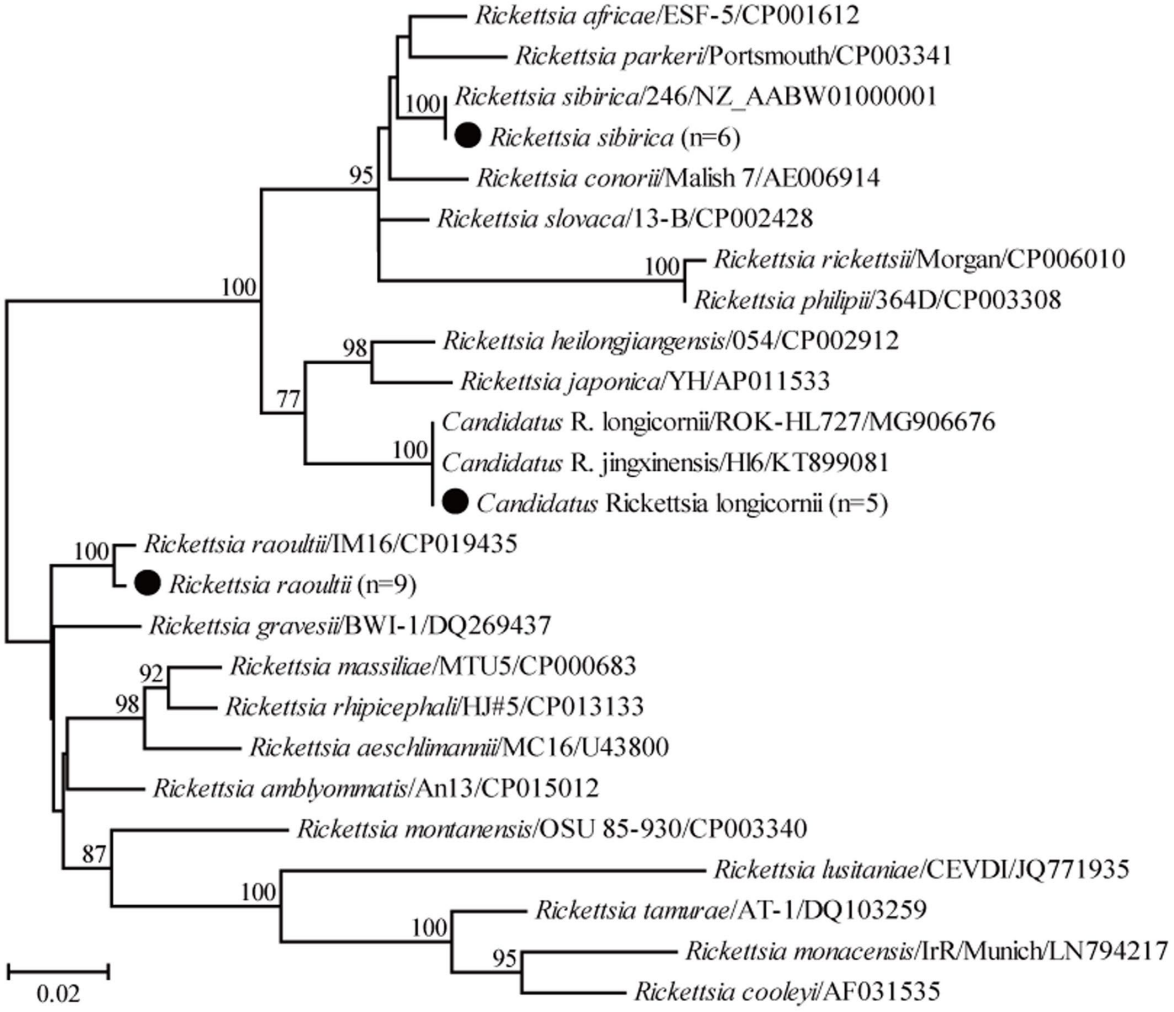
Phylogenetic tree based on the partial *ompA* gene sequences of the genus *Rickettsia*. Numbers at each node indicate bootstrap values. The tree was mid-point rooted for clarity, and the scale bar represents the number of nucleotide substitutions per site. The representative sequence obtained in this study was used to reconstruct the tree and is marked with circles.

For the genus *Rickettsia*, seven partial *ompA* gene sequences of *R. raoultii* in this study shared 99.2–100% nucleotide identity with each other and 97.7–99.4% with known *R. raoultii* variants. In addition, three partial *ompA* gene sequences of *R. sibirica* in this study shared 98.1–100% nucleotide identity with the known ones of *R. sibirica* variants. Another three partial *ompA* gene sequences of *Candidatus* R. longicornii in this study shared 98.8–100% nucleotide identity with each other and 96.6–100% nucleotide identity with known *Candidatus* R. longicornii variants. Interestingly, the *ompA* gene sequence of isolate Weichang-Sd-87 had an insertion of three bases (GAC) compared to the other four isolates, the same as isolates CCBF8, GXS12, and GXS34 identified from rodents in Guangxi, China. In the phylogenetic tree, 19 *ompA* gene sequences were classified into three groups, corresponding to *R. raoultii*, *R. sibirica*, and *Candidatus* R. longicornii (Figure 4).

## Discussion

Rodents are the main hosts of many human pathogens and can indirectly transmit vector-borne intracellular bacteria to humans, such as Rickettsiales and *Bartonella* (Han et al., 2015). The Daurian ground squirrel is a wild rodent species mainly distributed in northern China, Mongolia, and Russia and is a host of *Yersinia pestis* (Zhou et al., 2004a,b; Tian, 2018). Daurian ground squirrels frequently come into contact with herders, which can increase the risk of plague transmission in humans. To date, the diversity of vector-borne intracellular bacterial pathogens carried by Daurian ground squirrels has been unclear, although the ground squirrels are known to be hosts of ticks. In this study, eight intracellular bacterial species were discovered, namely two *Anaplasma* species, three *Rickettsia* species, and three *Bartonella* species. Specifically, these eight species were *A. capra*, *Candidatus* A. cinensis, *R. raoultii*, *R. sibirica*, *Candidatus* R. longicornii, *B. washoensis*, *B. grahamii*, and *B. jaculi*. Importantly, *A. capra*, *R. raoultii*, *R. sibirica*, *Candidatus* R. longicornii, *B. washoensis*, and *B. grahamii* are pathogenic to humans, suggesting potential threats to public health. In this study, a low rate of co-infection between *B. grahamii* and *R. raoultii* was observed. In a previous study, co-infection of *Rickettsia* and *Bartonella* was found to be prevalent in rodents in Vietnam (Anh et al., 2021), although the bacterial species within *Rickettsia* and *Bartonella* were not identified. All these results imply that the co-infection of *Rickettsia* and *Bartonella* in rodents does not interfere with each other. Our results give important insights into the distribution of these vector-borne pathogens and the genetic diversity of vector-borne pathogens in Daurian ground squirrels in the local area.

To date, *Rickettsia* has been found in many rodent species, even in single rodent species collected from a single location (Schex et al., 2011; Kuo et al., 2015; Lu et al., 2019). In previous studies, *R. heilongjiangensis*, *R. japonica*, and an unidentified *Rickettsia* species were detected in *Apodemus agrarius* in China (Lu et al., 2019); *R. felis* and *R. helvetica* were found in *A. flavicollis* in Germany (Schex et al., 2011); and seven *Rickettsia* species were identified in *Rattus losea* in Taiwan (Kuo et al., 2015). Similar to these results, three *Rickettsia* species, namely *R. raoultii*, *R. sibirica*, and *Candidatus* R. longicornii, were identified in Daurian ground squirrels in the present study, indicating that there is genetic diversity in *Rickettsia* hosted by a rodent species in a single location. In our previous studies, two uncultured *Rickettsia* species, *Candidatus* R. tarasevichiae, and *Candidatus* R. principis, were detected in ticks from the same county (Xue et al., 2023). However, neither was recovered in this study, although *Candidatus* R. tarasevichiae was detected in *R. norvegicus* and *Clethrionomys rufocanus* in Dashigou around Mudanjiang City, Heilongjiang Province, Northeast China (Yuan et al., 2021). This may be due to differences in sampling sites or because the Daurian ground squirrel is not the primary host. Our previous study also showed that at least three tick species, *Ixodes persulcatus*, *Dermacentor silvarum*, and *Haemaphysalis concinna*, were present in local areas, and *R. raoultii* has been identified in those ticks (Xue et al., 2023). However, *R. sibirica* and *Candidatus* R. longicornii were not identified, although *R. sibirica* has been detected in the same tick species from other parts of China (Zhao et al., 2021). Hence, ticks should be collected to determine their circulation and prevent further threats to human health.

As the main hosts, rodents are infected by many species within the genus *Bartonella*. Among the rodent-associated *Bartonella* species, eight detected in Daurian ground squirrels, including *B. rochalimae* and *B. washoensis*, are pathogenic to humans (Jian et al., 2022). In addition to *B. washoensis*, which was identified in Daurian ground squirrels in a previous study (Inoue et al., 2009; Li et al., 2023b), *B. grahamii* and *B. jaculi* were detected in Daurian ground squirrels in the present study. Combining the data from previous and our studies, four *Bartonella* species have been recovered in Daurian ground squirrels, indicating that this rodent species is an important host of *Bartonella*. *Bartonella jaculi* was first identified in *Jaculus orientalis* from Egypt (Sato et al., 2013) and then only detected in *Allactaga sibirica* from the Qaidam Basin of China (Rao et al., 2021). These results demonstrated the worldwide geographical distribution of *B. jaculi*, and it has a wide range of rodent hosts. In this study, *B. washoensis* was the predominant *Bartonella* species, followed by *B. grahamii*. Considering their pathogenicity to humans, the ectoparasites (including fleas, mites, lice, and ticks) of Daurian ground squirrels should be collected to determine those that can act as potential vectors of both *B. washoensis* and *B. grahamii* in future studies.

*Anaplasma capra* was first confirmed to be a human pathogen in 2015 (Li et al., 2015) and has been mainly identified in livestock based on sequences deposited in GenBank and previous studies (Guo et al., 2018; Amer et al., 2019; He et al., 2021; Remesar et al., 2022; Sahin et al., 2022). In this study, only one partial 16S rRNA sequence was obtained, and we conjectured that this may have resulted from a low bacterial load or DNA degradation by some enzymes in the host. Alternatively, rodents may not be a competent reservoir of *A. capra*. *Anaplasma platys* is considered a potential human pathogen because its DNA was detected in two female patients from Venezuela (Arraga-Alvarado et al., 2014). Unlike other *Anaplasma* species, *A. platys* showed a high level of great genetic diversity, manifested by the 16S rRNA gene forming a monophyletic group and the *gltA* and *groEL* genes forming polyphyletic groups (Ben Said et al., 2017; Guo et al., 2019; Nguyen et al., 2020; Zobba et al., 2022). In China, *A. platys*-like variants formed a lineage in the *groEL* tree, while they were classified into two lineages in the *gltA* tree (Guo et al., 2019). In Thailand, similar *A. platys*-like variants were also identified, and they clustered together with the above-mentioned variants in China in the *groEL* tree (Nguyen et al., 2020). At present, this lineage is named *Candidatus* A. cinensis. In addition, another two genetically related lineages of *A. platys*-like in ruminants from Tunisia were named *Candidatus* A. turritanum (Ben Said et al., 2017). Moreover, *Candidatus* A. camelii fell into the genetic diversity of *A. platys* and *A. platys*-like (Guo et al., 2019). In this study, *Candidatus* A. cinensis was identified in Daurian ground squirrels, and this is the first molecular evidence of its infection in rodents. In detail, the *gltA* gene sequences presented the closest genetic relationship with known sequences belonging to lineage 2 rather than those of lineage 1 from Guo et al. (2019), while the *groEL* gene sequences clustered together and formed one lineage. The data from this and previous studies suggest that *Candidatus* A. cinensis is widely distributed and infects a greater variety of potential hosts than suggested by previous studies (Ben Said et al., 2017; Guo et al., 2019; Nguyen et al., 2020). Therefore, its pathogenicity should be given more attention.

There were several limitations to this study. First, we did not check for the presence of blood-sucking vectors on the bodies of the ground squirrels in this study. Therefore, the real risks of the pathogens identified in this study to the local population and the association between the presence of vectors and infected Daurian ground squirrels could not be determined. As such, human cases should be identified in future studies to reveal the risks to the local population. Second, we did not record the age or gender of the captured rodents or the rodent numbers in each month because the aim of this study was to better understand the genetic diversity of vector-borne *Anaplasma*, *Rickettsia*, and *Bartonella*. Therefore, the assessment of the prevalence of each pathogen over time or across age groups or sexes of Daurian ground squirrels could not be determined. Moreover, rodent samples were collected from one sampling site for this study; therefore, potential spatial differences could not be determined, resulting in an incomplete picture of the intracellular bacterial pathogens in Daurian ground squirrels.

In summary, eight vector-borne bacterial species, including six that are pathogenic to humans, were molecularly identified in Daurian ground squirrels. Our results contributed to the characterization of the genetic diversity of bacterial species associated with Daurian ground squirrels, such as *Rickettsia*, *Anaplasma*, and *Bartonella*. Our results also provided evidence that Daurian ground squirrels were potential bacterial hosts. In addition, the results suggested that the pathogens could pose threats to public health in the local population. Considering that there could be an indirect transmission of the above-mentioned bacteria from Daurian ground squirrels to humans, potential vectors should be identified in future studies.

## Data availability statement

The datasets presented in this study can be found in online repositories. The nucleotide sequences recovered in this study were deposited in GenBank (https://www.ncbi.nlm.nih.gov/genbank) under the accession numbers OR976065 and OR988094–OR988128.

## Ethics statement

The animal study was approved by the ethical committee of Chengde Medical University. The study was conducted in accordance with the local legislation and institutional requirements.

## Author contributions

JX: Writing – original draft, Data curation, Formal analysis, Investigation. S-SC: Data curation, Investigation, Methodology, Writing – review & editing. Z-YX: Investigation, Resources, Writing – review & editing. F-NW: Investigation, Writing – review & editing. JW: Funding acquisition, Resources, Writing – review & editing. DD: Investigation, Resources, Writing – review & editing. LD: Supervision, Validation, Writing – review & editing. G-CX: Writing – review & editing. W-PG: Conceptualization, Funding acquisition, Methodology, Project administration, Supervision, Writing – original draft, Writing – review & editing.
