# Supplementary material for: Anaplasma, Bartonella, and Rickettsia infections in Daurian ground squirrels (Spermophilus dauricus), Hebei, China
Source: Front Microbiol. 2024 Mar 28;15:1359797. doi: 10.3389/fmicb.2024.1359797 (PMC11007220; doi:10.3389/fmicb.2024.1359797)
Supplement: Supplementary file 1 [file Table_1.DOCX]

Table S1 GenBank accession numbers of sequences obtained in this study.

|  | **Gene** | **Genbank numbers** | **Bacterial strain** |
| --- | --- | --- | --- |
| 1 | 16S rRNA | OR976065 | *Anaplasma capra* isolate Weichang-SPS192 |
| 2 | *gltA* | OR988094 | *Candidatus* Anaplasma cinensis isolate Weichang-SP112 |
| 3 | *gltA* | OR988095 | *Candidatus* Anaplasma cinensis isolate Weichang-SP134 |
| 4 | *gltA* | OR988096 | *Candidatus* Anaplasma cinensis isolate Weichang-SP135 |
| 5 | *gltA* | OR988097 | *Candidatus* Anaplasma cinensis isolate Weichang-SP137 |
| 6 | *gltA* | OR988098 | *Candidatus* Anaplasma cinensis isolate Weichang-SP139 |
| 7 | *gltA* | OR988099 | *Candidatus* Anaplasma cinensis isolate Weichang-SP169 |
| 8 | *groEL* | OR988100 | *Candidatus* Anaplasma cinensis isolate Weichang-SP112 |
| 9 | *gltA* | OR988101 | *Bartonella washoeensis* isolate Weichang-SP109 |
| 10 | *gltA* | OR988102 | *Bartonella washoeensis* isolate Weichang-SP143 |
| 11 | *gltA* | OR988103 | *Bartonella washoeensis* isolate Weichang-SP174 |
| 12 | *gltA* | OR988104 | *Bartonella washoeensis* isolate Weichang-SP187 |
| 13 | *gltA* | OR988105 | *Bartonella washoeensis* isolate Weichang-SPS121 |
| 14 | *gltA* | OR988106 | *Bartonella washoeensis* isolate Weichang-SPS128 |
| 15 | *gltA* | OR988107 | *Bartonella washoeensis* isolate Weichang-SPS140 |
| 16 | *gltA* | OR988108 | *Bartonella washoeensis* isolate Weichang-SPS150 |
| 17 | *gltA* | OR988109 | *Bartonella washoeensis* isolate Weichang-SPS164 |
| 18 | *gltA* | OR988110 | *Bartonella washoeensis* isolate Weichang-SPS184 |
| 19 | *gltA* | OR988111 | *Bartonella grahamii* isolate Weichang-SP1113 |
| 20 | *gltA* | OR988112 | *Bartonella grahamii* isolate Weichang-SP1126 |
| 21 | *gltA* | OR988113 | *Bartonella grahamii* isolate Weichang-SPS1135 |
| 22 | *gltA* | OR988114 | *Bartonella jaculi* isolate Weichang-SPS1109 |
| 23 | *gltA* | OR988115 | *Bartonella jaculi* isolate Weichang-SPS1143 |
| 24 | *ompA* | OR988116 | *Candidatus* Rickettsia longicornii isolate Weichang-SP120 |
| 25 | *ompA* | OR988117 | *Candidatus* Rickettsia longicornii isolate Weichang-SP65 |
| 26 | *ompA* | OR988118 | *Candidatus* Rickettsia longicornii isolate Weichang-SP87 |
| 27 | *ompA* | OR988119 | *Rickettsia raoultii* isolate Weichang-SP7 |
| 28 | *ompA* | OR988120 | *Rickettsia raoultii* isolate Weichang-SP75 |
| 29 | *ompA* | OR988121 | *Rickettsia raoultii* isolate Weichang-SP111 |
| 30 | *ompA* | OR988122 | *Rickettsia raoultii* isolate Weichang-SP119 |
| 31 | *ompA* | OR988123 | *Rickettsia raoultii* isolate Weichang-SP121 |
| 32 | *ompA* | OR988124 | *Rickettsia raoultii* isolate Weichang-SP122 |
| 33 | *ompA* | OR988125 | *Rickettsia sibirica* isolate SP72 |
| 34 | *ompA* | OR988126 | *Rickettsia sibirica* isolate SP76 |
| 35 | *ompA* | OR988127 | *Rickettsia sibirica* isolate SP98 |
| 36 | *ompA* | OR988128 | *Rickettsia sibirica* isolate SP126 |
